# Exploring the frontier of microbiome biomarker discovery with artificial intelligence

**DOI:** 10.1093/nsr/nwae325

**Published:** 2024-09-13

**Authors:** Liwen Xiao, Fangqing Zhao

**Affiliations:** Institute of Zoology, Chinese Academy of Sciences, China; Institute of Zoology, Chinese Academy of Sciences, China; Key Laboratory of Systems Health Science of Zhejiang Province, School of Life Science, Hangzhou Institute for Advanced Study, University of Chinese Academy of Sciences, China

**Figure 1.**
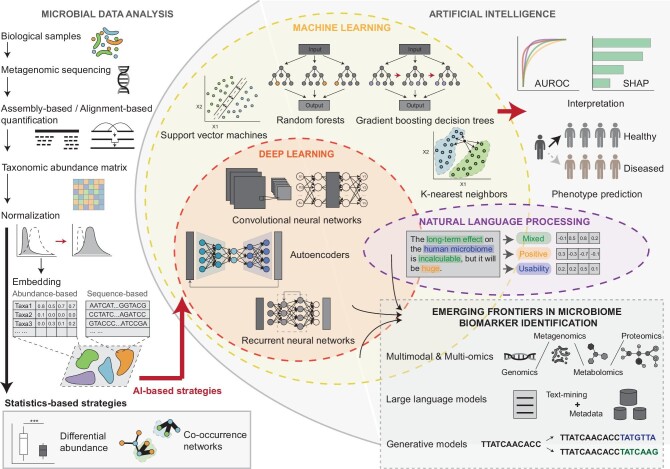
Microbiome biomarker identification based on traditional strategy and AI strategy. Metagenomic sequencing data analysis involves several key steps, including sample collection and processing, sequencing, quality control, quantification, taxonomic classification and normalization. Traditional biomarker identification methods rely on statistical techniques (e.g. *t*-test, ANOVA, network analysis algorithms) to find differentially abundant taxa or disturbed network modules. AI-based approaches offer refined frameworks for feature representation, model fine-tuning, prediction and interpretation. These approaches leverage various machine-learning and deep-learning techniques, such as random forests, support vector machines and neural networks, that show promising performance for future applications in personalized medicine and microbiome-based diagnostics. The integration of AI algorithms with cutting-edge sequencing technologies, such as multimodal/multi-omics integration, large language models and generative models, will significantly enhance the efficiency and precision of microbiome biomarker identification.

The human microbiome refers to the entirety of bacteria, archaea, fungi and viruses in the human body, which are extensively linked to human health and play crucial roles in the occurrence and progression of various diseases, including cancers. Due to their feasible sampling and clear representation, components of the microbiome, particularly bacteria, act as disease signatures or ‘biomarkers’ for predicting and diagnosing various diseases [1]. Traditional biomarker identification strategies rely on the differential abundance of specific taxa. Microbiome sequencing data result in a taxonomy abundance matrix in which the rows represent taxa and the columns represent samples [2]. Specific statistical tests are used to determine which taxa are significantly different between groups, with the most significant taxa being considered biomarkers related to particular biological conditions such as diseases or interventions (Fig. 1).

Nevertheless, robust microbial biomarker identification faces several challenges. Genetic and external factors, including ethnicity, diet, living environments and varying experimental conditions, can confound microbial sequencing and annotation, introducing unwanted biases and variations in results. Additionally, intrinsic features of microbial data, such as sparsity and compositionality, pose challenges for accurately identifying differential abundance bacteria [3–5]. Recent studies have attempted to address these issues by employing various approaches to correct and remove variations in microbial abundance quantification. For instance, the spike-in approach involves adding known quantities of exogenous microbial species to samples before sequencing, providing internal standards for absolute quantification. Alternatively, post-hoc statistical methods such as ANOVA-like differential expression (ALDEx2) or analysis of the compositions of microbiomes with bias correction (ANCOM-BC) use computational techniques to infer absolute abundances from relative abundance data [6,7]. While these methods enhance quantification accuracy, they also affect downstream analyses. For instance, spike-in taxa may interact with the native microbiome or have different extraction and amplification efficiencies, potentially altering community composition. Statistical transformations, although powerful, rely on assumptions about data distribution and compositionality that may not always hold true in complex microbial ecosystems. Recent studies that reanalysed differentially abundant taxa across multiple data sets using various strategies have found that no single statistical approach consistently performs well across multiple data sets [8,9]. This finding suggests that microbiome abundance data from sequencing may obscure genuine differences, necessitating the careful selection of current statistical strategies during biomarker identification. To address these issues, researchers are exploring alternative strategies, such as analysing microbiome co-occurrence [10]. By treating the microbiome as a cohesive community and using co-occurrence networks to identify significantly disturbed modules between populations, the efficiency and robustness of biomarker identification can be improved [2]. Nonetheless, these methods still face challenges, as microbiome co-occurrence may be influenced by the selection of different network construction methods.

Recently, artificial intelligence (AI) has offered a novel approach to tackling complex problems. Compared with traditional methodologies, AI provides significant advantages in pattern recognition, natural language processing (NLP), causal inference and outcome prediction. These capabilities make AI highly suitable for processing complex and high-dimensional microbiome data. Previous studies have shown that AI performs well in biomarker identification and phenotype prediction [3,11]. A typical machine-learning (ML) algorithm, a prominent branch of AI, comprises two phases: during the training phase, a robust model is constructed using labeled data, while the prediction phase uses this trained model to predict targets with unlabeled data (Fig. 1). By translating complex metagenomic data into ML-compatible formats and applying appropriate algorithms, researchers can uncover taxa or functional elements such as genes or bacteria-related metabolites that significantly contribute to specific phenotypes or conditions, helping to predict disease outcomes or responses to interventions [12]. Beyond model accuracy, interpretability is critical in understanding how these biomarkers influence predictions. Interpretability tools such as SHAP (SHapley Additive exPlanations) make AI models more transparent by revealing the impact of specific features on disease prediction. Performance metrics such as area under the receiver operating characteristic curve (AUROC) for classification tasks, mean squared error for regression problems, and F1-score for precision and recall balance help evaluate the accuracy of an algorithm in biomarker discovery and the prediction of microbial dynamics [11]. These metrics, combined with interpretability methods, ensure that AI-driven insights are both accurate and explainable, which is crucial for the advancement of microbiome-based biomarker research (Fig. 1). Support vector machines (SVM) and partial least squares discriminant analysis (PLS-DA) are both classic supervised ML algorithms. They implement different strategies to identify features that are most correlated with the host phenotypes, such as disease states, treatment responses or genetic variations. Other methods, such as the least absolute shrinkage and selection operator (LASSO) and ridge regression, have also demonstrated effectiveness in feature selection and prediction tasks. Compared with these algorithms, ensemble learning methods, particularly random forests (RFs) and gradient-boosting decision trees (GBDTs), have demonstrated promising results due to their ability to handle high-dimensional data and capability to capture complex interactions between microbial features. Recent studies have applied these models to disease evaluation and dynamics prediction, achieving AUROC scores of 0.7–0.9 across various diseases and physiological states [13–17]. While supervised learning methods dominate current research, unsupervised learning algorithms, such as clustering and dimensionality reduction techniques, are gaining traction. These methods can reveal inherent patterns in microbiome data without prior labeling, potentially uncovering novel microbial associations or subgroups within diseases.

Despite their superior interpretability, classic ML methods often find it difficult to extract latent patterns that are hidden within complex microbiome data sets and generalize them to extensive data sets. Compared with that, deep learning (DL), a subset of ML, employs neural networks (NNs) with multiple layers to model complex patterns from input data, potentially identifying microbiome markers more accurately by integrating large data sets [3]. Numerous studies have developed tools to enhance biomarker discovery efficiency based on NN frameworks. The fundamental approach involves using k-mer-based embedding or other representations (e.g. one-hot encoding and phylogenetic tree-based encoding) to encode taxonomic reads or the relationships between them. By utilizing these biologically informed representations, the hidden nodes in the NNs can potentially correspond to meaningful biological features or taxonomic relationships. This approach not only enhances the performance of the model in biomarker discovery, but also improves interpretability, allowing researchers to gain insights into the underlying mechanisms that are driving the observed patterns in the microbiome data. NNs encompass various architectures that are tailored to different situations. Convolutional neural networks (CNNs), for instance, contain convolutional layers that are specialized for processing grid-like data such as images and have been adapted for microbiome biomarker identification. Recurrent neural networks (RNNs), on the other hand, are well suited for sequential data, as they maintain hidden states and incorporate recurrent connections to capture information from previous inputs, making them ideal for tasks in which order matters. Autoencoders (AEs) are another popular NNs framework that compresses input data into a lower-dimensional latent-space representation to extract salient features. This architecture is particularly suitable for unsupervised learning tasks, such as predicting microbiome composition alteration during disease or dietary intervention and identifying robust biomarkers. Recently, these models and their extensions have been used to predict host phenotypes, disease progression and microbial dynamics, showing promising results compared with traditional methods [18–20]. The choice of AI algorithm often depends on the specific research objectives and the characteristics of the microbiome data. 16S rRNA amplicon sequencing data may benefit more from unsupervised techniques that can extract meaningful taxonomic representations, while shotgun metagenomic sequencing data may be better suited for supervised methods that can uncover functional relationships. Considering their advantages, contemporary studies typically employ multiple AI algorithms for disease prediction to enhance the interpretability and robustness of microbiome-based insights across diverse data sets. However, this observation also suggests potential biases in different studies during sampling and sequencing, emphasizing the importance of input metagenomic data preprocessing and an embedding strategy during model construction.

While a variety of AI algorithms have demonstrated promising results in microbiome biomarker identification, several emerging frontiers hold significant potential for advancing the field. One promising area is the integration of multimodal data, going beyond just taxonomic and functional profiles of the microbiome. By combining microbiome data with other omics, such as host genetics, transcriptomics, proteomics and metabolomics, AI models can uncover complex systems-level relationships that drive disease phenotypes. This approach reveals novel microbial biomarkers that are predictive of clinical outcomes while also providing mechanistic explanations for their associations. A notable advancement in AI is the application of NLP—particularly large language models (LLMs). By utilizing a transformer architecture with attention mechanisms, LLMs can dynamically weigh the importance of different parts of the input data, such as text and metadata, capturing long-range dependencies and complex relationships within the data. This enhances their ability to understand sequence context and integrate knowledge from diverse sources, improving their potential to identify biomarkers, combining them with scientific literature, patient records and microbiome-related annotations. Although their application in microbiome research is still emerging, LLMs hold great potential to analyse large-scale textual data to generate new hypotheses, prioritize biomarker candidates and synthesize existing knowledge. For example, LLMs can predict microbial functions and disease associations by processing metadata from clinical trials, experimental annotations or textual descriptions of microbial traits. These context-aware capabilities and emergent properties, which are highly relevant for understanding microbial communities, potentially offer a revolutionary approach to disease prediction methodologies. Another exciting frontier is the application of generative AI models such as generative adversarial networks (GANs) and variational autoencoders (VAEs). In the context of microbiomes, these models could create diverse in silico microbial communities, allowing researchers to test the robustness of biomarker identification algorithms and generate synthetic data sets for model training when real-world data are scarce. Such capabilities can accelerate the development and validation of AI-powered biomarker detection tools (Fig. 1). Looking ahead, the integration of long-read sequencing technologies with advanced AI methods also holds immense potential. The ability of long-read platforms to capture full-length 16S rRNA genes, reconstruct complete microbial genomes and provide strain-level resolution can unlock new frontiers in microbiome biomarker identification.

AI algorithms offer notable advantages for biomarker identification in metagenomic data sets; however, it is important to acknowledge the substantial challenges that this approach faces. One major issue is the acquisition of metadata, particularly clinical information, which often involves personal privacy concerns and is difficult to access. This limitation significantly hinders comprehensive evaluation and clear interpretation of the results. Given that AI models are frequently characterized as ‘black boxes’, facilitating access to critical metadata becomes particularly crucial for enhancing model interpretability. Another challenge is the intensive computational cost associated with training large AI models, especially when integrating extensive and complex data sets. A thoughtful balance between model performance and computational efficiency must be carefully considered. Furthermore, the selection of appropriate normalization and embedding strategies plays a crucial role in feature selection and model construction, which ensures the generation of unbiased and accurate results. Addressing these challenges necessitates significant efforts to integrate AI effectively into microbiome research and clinical applications. By improving these methods and tackling these challenges, AI can become a powerful tool to enhance our understanding of microbiome-related health and disease, paving the way for microbiome-based diagnosis and therapeutic strategies.
